# Transcriptome RNA Sequencing Reveals That Circular RNAs Are Abundantly Expressed in Embryonic Breast Muscle of Duck

**DOI:** 10.3390/vetsci10020075

**Published:** 2023-01-19

**Authors:** Jing Liu, Shuibing Liu, Wentao Zhang, Xiaolong Hu, Huirong Mao, Sanfeng Liu, Biao Chen

**Affiliations:** 1 College of Animal Science and Technology, Jiangxi Agricultural University, Nanchang 330045, China; 2 Poultry Institute, Jiangxi Agricultural University, Nanchang 330045, China

**Keywords:** duck, circular RNA, *GAS2*, skeletal muscle, proliferation

## Abstract

**Simple Summary:**

Research into the genetic mechanisms of skeletal muscle development in ducks is of great significance to the breeding and industrialization of indigenous ducks in China. This study examined the crucial candidates during two developmental stages of duck embryonic breast muscles by circular RNA sequencing. There were 16,622 circular RNAs detected between embryonic day 13 (E13) and embryonic day 19 (E19), of which 260 showed differential expression. Additionally, we examined the protein–protein interaction (PPI) with the parental genes of the differentially expressed circular RNAs, as well as the enriched GO terms and KEGG pathways. In addition, we selected one circular transcript of the growth arrest-specific gene 2 (*GAS2*), or circGAS2-2, to perform functional validations in duck embryonic myoblasts. The results showed that circGAS2-2 accelerated cell cycling and promoted the proliferation of myoblasts. Our results supplement the duck circular RNA database and demonstrate that circular RNAs are abundantly and differentially expressed during duck skeletal muscle development.

**Abstract:**

Circular RNAs are widespread in various species and have important roles in myogenesis. However, the circular RNAs involved in breast muscle development in ducks have not yet been studied. Here, to identify circular RNAs during duck skeletal muscle development, three pectorales from Shan Ma ducks at E13 and E19, which represent undifferentiated and differentiated myoblasts, respectively, were collected and subjected to RNA sequencing. A total of 16,622 circular RNAs were identified, of which approximately 80% were exonic circular RNAs and 260 were markedly differentially expressed between E19 and E13. The parental genes of the differentially expressed circular RNAs were significantly enriched in muscle-related biological processes. Moreover, we found that the overexpression of circGAS2-2 promoted cell cycle progression and increased the proliferation viability of duck primary myoblasts; conversely, knockdown of circGAS2-2 retarded the cell cycle and reduced the proliferation viability of myoblasts. Taken together, our results demonstrate that circular RNAs are widespread and variously expressed during the development of duck skeletal muscle and that circGAS2-2 is involved in the regulation of myogenesis.

## 1. Introduction

China is the biggest duck meat producer in the world. The consumption of poultry meat in China has significantly increased as a result of African swine fever (http://data.stats.gov.cn/, accessed on 1 September 2022). The development of poultry skeletal muscle is influenced by many factors, including genetics, diet, and the environment [1,2]. Myogenesis can be divided into the following several stages: somite differentiation into myoblasts; myoblasts’ migration and expression of particular myogenic transcription factors (MTFs); myoblasts’ differentiation into myotubes under the influence of MTFs [3]. The process of myoblast differentiation is accompanied by cell cycle pausing and the adherence and fusion of myoblasts [4]. This process is regulated by multiple genetic factors, including transcription factors, microRNAs (miRNAs), and long non-coding RNAs [5,6,7,8]. For this reason, it is important to conduct research on the genetic mechanisms behind the development of skeletal muscle in ducks in order to breed and industrialize the indigenous ducks of China.

Circular RNAs are non-coding RNAs distinguishable from linear RNAs by their covalent head-to-tail structures [9,10]. Circular RNAs were previously considered to be useless products of the transcriptome but are now known to be gene regulators and to possess protein-coding abilities [11,12,13,14,15]. Circular RNAs are formed by non-canonical splicing (back-splicing) of precursor mRNAs [16]. This alternative back-splicing enables various circular RNA types, including exonic circular RNAs (circRNAs), intronic circular RNAs (ciRNAs), and exon–intron circular RNAs, to be generated from the same gene [12]. Previous studies have shown that circular RNAs in the nucleus could act as gene regulators by modulating transcription, alternative splicing, and chromatin looping [17,18]. Circular RNAs can also serve as miRNA sponges and function as competing endogenous RNAs [9,19], as well as participate in various biological processes by binding various proteins [20,21].

The transcriptomes of many tissues and species contain circular RNAs, which play key roles in cellular and physiological functions [22,23,24,25]. They have been found in skeletal muscle at different developmental stages in various species [26,27,28], and they regulate myoblasts’ proliferation and differentiation, mainly through acting as miRNA scaffolds [29]. However, there have been no reports on the formation and expression of circular RNAs in duck skeletal muscle. A genome-wide circular RNA sequencing study was performed on Shan Ma duck embryonic breast muscle at embryonic days 13 (E13) and 19 (E19). In E13, the majority of myoblasts in the skeletal muscle remained undifferentiated. Meanwhile, almost all of the myoblasts had differentiated and fused to myotubes in E19, and the breast muscle weight remained almost the same from E19 to hatching [30]. We analyzed the molecular properties and potential functions of these circular RNAs and explored their functions in duck myoblasts.

## 2. Materials and Methods

### 2.1. Samples for Circular RNA Sequencing

As previously described, 180 hatching eggs of Shan Ma ducks with the same genetic background were obtained from the Tianyun duck breeding farm (Jiangxi, China) and incubated at 37.2 °C with a relative humidity of 60 ± 10% [31]. Breast muscle samples and livers of 12 ducks were sacrificed every 3 days from E10 to P1 (E10, E13, E16, E19, E22, and P1). The duck embryos were extracted from the eggs with tweezers. The morphology of all of the ducks was checked to ensure the correct developmental stage. The pectoral and leg muscles were sampled after the skin and feathers were eliminated. As soon as the tissues were sampled, they were frozen in liquid nitrogen and kept at −80 °C. Using PCR amplification of the chromo-helicase-DNA-binding 1 (*CHD1*) gene, which is found on the Z and W chromosomes, the embryos were identified according to their sex [32]. The circRNA sequencing was performed with three pectorales from female ducks at E13 and E19, respectively.

### 2.2. Library Construction and Sequencing

Following the manufacturer’s instructions, the total RNA of the six samples was extracted and purified with TRIzol reagent (Invitrogen, Carlsbad, CA, USA), and the amounts and purities of the RNAs were quantified (ND-1000, NanoDrop, Wilmington, DE, USA). The RNA integrity was assessed (Agilent 2100, Agilent Technologies, Palo Alto, CA, USA) with an RNA integrity number of > 7.0. In order to deplete the rRNA, approximately 5 ug of the total RNA was used, following the instructions from the Ribo-Zero^TM^ rRNA Removal Kit (Illumina, San Diego, CA, USA). The remaining RNA was treated with RNase R (Epicentre, Madison, WI, USA) to remove the linear RNAs and enrich the circRNAs. As a result of fragmentation and reverse transcription of the enriched circRNAs, cDNA was synthesized, and U-labeled second-stranded DNAs were synthesized with DNA polymerase I, RNase H, and dUTP. To prepare the blunt ends of each strand for ligation to the indexed adapters, an A-base was added. The fragments were ligated to single- or dual-index adapters, and AMPureXP beads were used for the size selection. A PCR amplification of the UDG-treated second-stranded DNAs was carried out after the products had been heat-labilely treated with the enzyme. In the final cDNA library, the average insert size was 300 ± 50 bp. In the final step, paired-end sequencing was performed on an Illumina HiSeq 4000 platform (LC Bio, Hangzhou, Zhejiang, China) using the vendor’s recommended protocols.

### 2.3. Annotation of Duck circRNAs and Differential Expression Analysis between E13 and E19

The reads with adaptors, low-quality bases, or undetermined bases were removed with Cutadapt [33]. The quality of the sequence was verified using FastQC (http://www.bioinformatics.babraham.ac.uk/projects/fastqc/, accessed on 1 February 2020). The sequencing reads were mapped to the genome of Anas platyrhynchos (http://asia.ensembl.org/Anas_platyrhynchos/Info/Index, accessed on 3 February 2020) using Bowtie 2 and TopHat 2 [34,35]. The mapping of the remaining reads was performed using TopHat-Fusion [36]. The mapped reads were assembled into circular RNAs using CIRCExplorer2 and CIRI. TopHat-Fusion was then used to identify the back-splicing reads among the unmapped reads. All of the samples produced distinct circular RNAs [37,38,39].

The relative expression levels of the circRNAs were represented by spliced reads per billion mapping (SRPBM). The circular RNA expression levels from the different groups (E13 and E19) were calculated using in-house scripts. The R package was used for the comparisons, with a |log_2_fold change| of ≥ 1 and a *p*-value of ≤ 0.05 regarded as indicating differential expression [40].

### 2.4. Enrichment Analysis of the Differentially Expressed circRNAs

Using the DAVID functional annotation tool [41], we performed a pathway enrichment for the parental genes of the differentially expressed circRNAs using Gene Ontology (GO) and the Kyoto Encyclopedia of Genes and Genomes (KEGG). The parental genes were also subjected to gene network analyses using STRING v11.0 and Cytoscape v3.7.2 [42].

### 2.5. cDNA Synthesis and qRT-PCR

The Monad MonScript^TM^ All-in-One Kit (Biopro, Shanghai, China) with DNase was used to reverse-transcribe the total RNA obtained from the animal tissues or cell lines. The synthesized cDNA samples were then diluted in nuclease-free water at a ratio of 1:4 for qPCR. The relative mRNA or circular RNA expression were determined by qRT-PCR in a final volume of 20 µL with 2 × T5 Fast qPCR Mix from TsingKe Bio (Beijing, China). The internal control was the duck *GAPDH* gene. In order to identify the circRNAs, qRT-PCR was performed on the RNA samples with and without digestion by RNase R. The qRT-PCR program was run by an ABI Q5 system (Thermo Fisher, Waltham, MA, USA) using the following procedure: 95  °C for 3 min; 40 cycles of 95  °C for 10  s; annealing temperature for 1 min; collection of fluorescence from 65 to 95 °C. Each sample was examined with three technical repeats, and the expression levels were calculated using the comparative 2^−∆∆Ct^ method and presented as means ± S.E.M. A Student’s t-test and an one-way ANOVA test were used to compare the expression levels among the different groups. Table S1 lists all of the primers utilized in the current study.

### 2.6. Validation of circRNAs

The circRNAs were validated by RT-PCR with divergent and convergent primers using cDNA or gDNA as the templates. The products were gel purified and cloned with a pClone007 vector kit (TsingKe). The DNASTAR (SeqMan) software (http://www.dnastar.com/, accessed on 3 January 2021) was used to align and analyze the results of positive clones sent to TsingKe for Sanger sequencing.

### 2.7. Overexpression Plasmid Construction and siRNA Synthesis

For the construction of the circGAS2-2 overexpression vector, exons 5, 6, and 7 of the duck *GAS2* gene were amplified using cDNA from the pectorales and inserted into a pK25ssAAV-ciR vector (Geneseed, Guangzhou, China) between the *EcoRI* and *BamHI* restriction sites. Geneseed was used to design and synthesize the circGAS2-2 siRNA based on the sequence listed in Table S1.

### 2.8. Cell Isolation, Culture, and Electroporation

Duck primary myoblasts were extracted from the pectorales of E13 Shan Ma duck embryos (Tianyun). The breasts of E13 ducks were collected and washed in pre-cooled phosphate-buffered saline with 0.5% penicillin/streptomycin (Invitrogen). The muscle samples were cut into pieces using scissors and then trypsinized (Gibco, Grand Island, NY, USA) at 37 °C for 40 min after the skin and bones had been removed. The digestion was terminated with fetal bovine serum (FBS, Gibco, Grand Island, NY, USA). The mixture was filtered using a 40-μm cell strainer (Sangon, Shanghai, China) and centrifuged at 1100 rpm for 5 min. After serial plating, the cells were grown in complete Dulbecco’s modified Eagle medium (Gibco, Grand Island, NY, USA) with 15% FBS and 0.2% penicillin/streptomycin and incubated at 37 °C in a 5% CO_2_ humidified atmosphere. The myoblasts were induced to differentiate using 0.5% FBS. A total of 5 µg of DNA plasmid and 5 nmol of siRNA or siRNA NC were mixed with 5 × 10^6^ myoblasts in 200 μL of Entranster-E electroporation solution (Engreen, Beijing, China), and cell electroporation was performed on a BTX ECM2001 (BTX, San Diego, CA, USA).

### 2.9. Flow Cytometry of Cell Cycle Analysis

A total of 5 × 10^6^ myoblasts were collected and electroporated with an overexpression plasmid or vector control and siRNA or siRNA NC. Then, the cells were seeded in a six-well plate. After 48 h, the myoblasts were collected in 70% ethanol and stored overnight at −20 °C, followed by incubation with 50 mg/mL of propidium iodide (Sigma, St Louis, MO, USA) containing 10 mg/mL of RNase A (Takara, Otsu, Shiga, Japan) and 0.2% (*v*/*v*) of Triton X-100 (Sigma) at 4 °C for 30 min. Based on the DNA content in the different phases (G1, S, and G2), the results of the BD AccuriC6 flow cytometer (BD, San Jose, CA, USA) were analyzed using Flow-Jo7.6 (https://www.flowjo.com/, accessed on 10 July 2020). The results were presented as means ± S.E.M. A Student’s t-test was used to compare the cell numbers among the different phases.

### 2.10. Cell-Counting Assay

After the cell electroporation, the myoblasts were seeded in a 96-well plate. Cell proliferation was detected at 12 h, 24 h, 36 h, 48 h, and 60 h using a TransDetect CCK kit (TransGen, Beijing, China), following the manufacturer’s instructions. Briefly, 10 µL of CCK solution was added to each well and incubated for 1.5 h at 37 °C with 5% CO_2_. Then, an Infinite 200 PRO plate reader (Tecan, Männedorf, Switzerland) was used to measure the absorbance at a wavelength of 450 nm.

## 3. Results

### 3.1. Overview of RNA Sequencing Data and Identification of Duck Circular RNA

To identify the circular RNAs in the duck muscle samples, we performed genome-wide circular RNA sequencing with RNase R digestion, followed by functional analysis in duck primary myoblasts. HPF/HPR primers were used to amplify the *CHD1* and distinguish the sex of the embryos (Figure S1). Breast muscle tissues from three female Shan Ma duck embryos at E13 and E19 were subjected to circular RNA sequencing after ribosomal RNA (rRNA) depletion and RNase R digestion.

**Figure 1:**
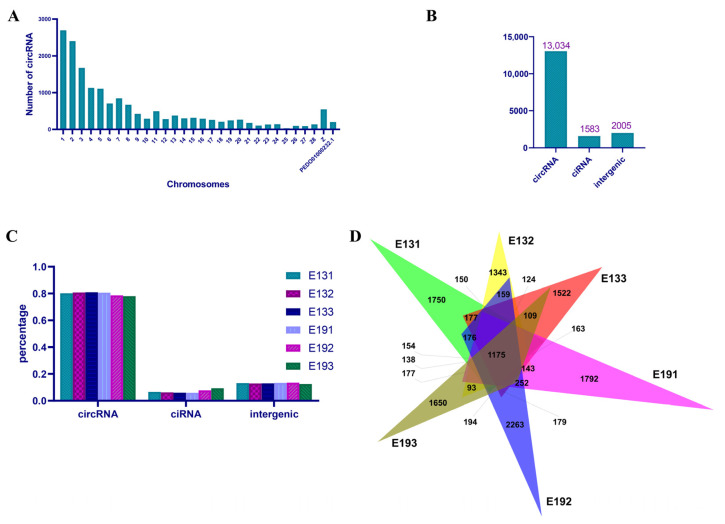
Circular RNA expression profiling. (**A**) Distribution of circular RNAs on duck chromosomes. (**B**) Numbers of different types of circular RNA. (**C**) Percentages of different types of circular RNA in each sample. circRNA, exonic circular RNA; ciRNA, intronic circular RNA; intergenic, circular RNA generated from intergenic sequence. (**D**) Venn diagrams of circular RNAs expressed in each sample.

A total of 290,131,458 reads were sequenced from all of the six samples (Table S2). After eliminating the low-quality reads and adaptors, 159,352,398 clean reads were mapped to the duck genome (Table S2). The unique mapped reads, multi-mapped reads, pair-end mapped reads, reads mapped to sense strands, and reads mapped to antisense strands are shown in Table S2. Then, the circular RNAs were identified using the following criteria: a mismatch of ≤ 2, back-spliced junction reads of ≥ 1, and a distance between two splice sites of < 100 kb. Using these criteria, a total of 19,886 circular RNAs, generated by 10,882 genes, were found among 4,284,753 candidate back-spliced junction reads (Table S3). Of these circular RNAs, 13,034 were circRNAs, 1583 were ciRNAs, and 2005 originated from intergenic sequences (Figure 1B). They were mostly found on chromosomes 1, 2, 3, 4, 5, and Z (Figure 1A). The predominant type of circular RNA in the duck skeletal muscle was circRNA (approximately 80%), while the ciRNAs and intergenic circular RNAs were the minor types (Figure 1C). A total of 1175 circular RNAs were detected in all of the six samples; 1663 circular RNAs were expressed in three of the E13 samples, and 1890 circular RNAs were found in all of the E19 samples (Figure 1D and Figure S2). Overall, the RNA sequencing data showed that circular RNAs are copiously expressed in duck embryonic skeletal muscle.

### 3.2. Identification of Differentially Expressed Circular RNAs

**Figure 2:**
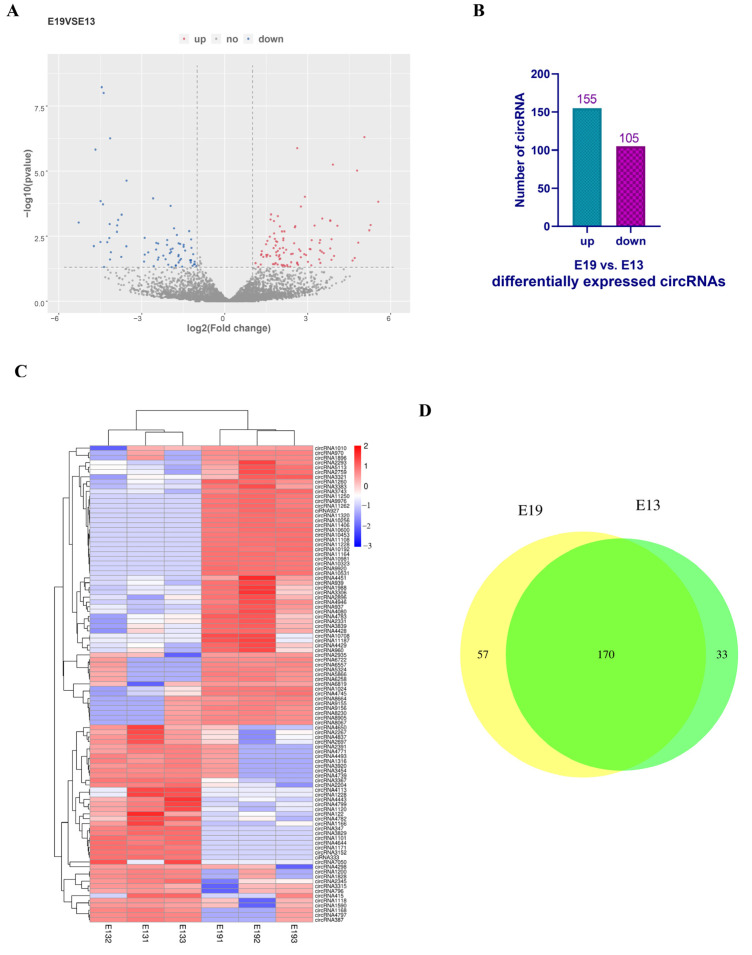
Circular RNAs are expressed differently in two stages of duck embryonic breast muscle development. (**A**) Volcano plot of circular RNAs expressed at E19 vs. E13. The red dots represent upregulated circular RNAs; the blue dots represent downregulated circular RNAs; the grey dots represent no difference in expression. (**B**) Numbers of upregulated and downregulated circular RNAs. (**C**) Heat map of the top 100 differentially expressed circular RNAs at E19 vs. E13 in the pectorales. (**D**) Venn diagrams of differentially expressed circular RNAs at E19 vs. E13.

To explore the potential functions of the circular RNAs expressed in the duck skeletal muscle, we performed a differential expression analysis using the following criteria: a |log_2_ fold change| of ≥ 1 and a *p*-value of ≤ 0.05 (Figure 2A). A total of 260 circular RNAs, of which 155 were upregulated and 105 were downregulated, were differentially expressed between the E19 group and the E13 group (Figure 2B). The top 100 differentially expressed circular RNAs were then clustered. The data were normalized by SRPBM. The results showed that the upregulated and downregulated circular RNAs were quite distinct between the E13 and E19 groups (Figure 2C). A total of 170 circular RNAs were expressed in both of the groups, of which 57 were specifically expressed in the E19 group and 33 were uniquely expressed in the E13 group (Figure 2D). The results demonstrate that duck embryonic breast muscle development is characterized by the differential expression of many circular RNAs.

### 3.3. Enrichment Analysis of the Differentially Expressed Circular RNAs

**Figure 3:**
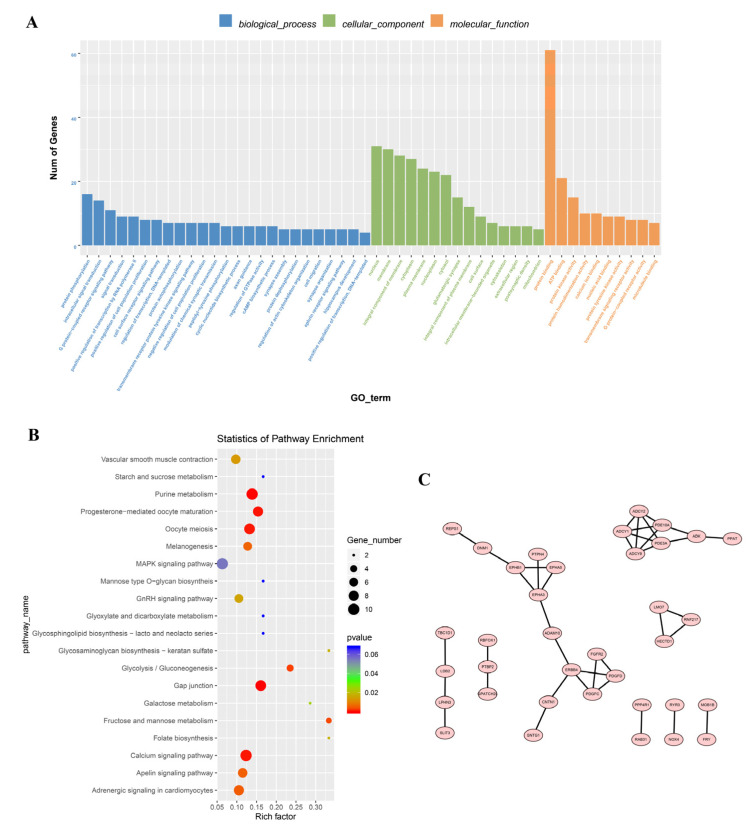
Enrichment of the differentially expressed circRNAs. (**A**) Classification of the parental genes of the differentially expressed circular RNAs based on GO functions. The top 10, 15, and 25 terms in molecular functions, cellular components, and biological processes are displayed, respectively. (**B**) The top 20 enriched KEGG pathways of the parental genes of the circular RNAs with differential expression. (**C**) PPI network for the parental genes of the differentially expressed circular RNAs.

Both circular RNAs and mRNAs originate from pre-mRNA splicing, and circular RNAs can regulate the expression of other transcripts of their parental genes. To predict the function of circular RNAs in embryonic skeletal muscle development, a Gene Ontology (GO) enrichment was performed for the parental genes of the differentially expressed circular RNAs in terms of cellular components, biological processes, and molecular functions. The parental genes were associated with 182 GO terms. Protein binding, ATP binding, protein kinase activity, nucleus, membrane, integral component of membrane, protein phosphorylation, intracellular signal transduction, and G protein-coupled receptor signaling were the most highly enriched GO terms (Figure 3A). The parental genes were also enriched in muscle-related GO terms, including regulation of skeletal muscle cell differentiation (*p* < 0.01) and cardiac muscle tissue regeneration (*p* < 0.01) (Table S4). In addition, a Kyoto Encyclopedia of Genes and Genomes (KEGG) analysis showed that the parental genes of the differentially expressed circular RNAs were significantly enriched in 16 signaling pathways, including gap junctions, the calcium signaling pathway, purine metabolism, progesterone-mediated oocyte maturation, adrenergic signaling in cardiomyocytes, vascular smooth muscle contraction, the GnRH signaling pathway, and cardiac muscle contractions (Figure 3B, Table S5). Furthermore, we performed a protein–protein interaction (PPI) analysis of the parental genes using STRING. A total of 36 proteins encoded by the parental genes of the differentially expressed circular RNAs interacted with other parental gene products, including some muscle-related proteins, such as the fibroblast growth factor receptor 2 (*FGFR2*), members of the adenylate cyclase and EPH families, and erythropoietin-producing hepatoma (*RBFOX1*) (Figure 3C). These results indicate that differentially expressed circular RNAs are associated with muscle development.

### 3.4. Verification of the Differential Expression Circular RNAs

**Figure 4:**
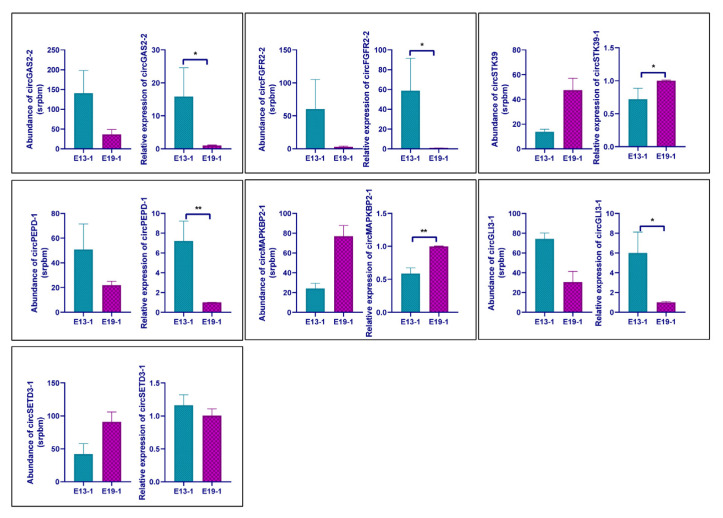
qRT-PCR verification of the differentially expressed circRNAs. The values are shown as means ± SEM. * *p* < 0.05; ** *p* < 0.01.

To validate the differential expression of the circular RNAs identified in this study, seven circRNAs were randomly selected for qRT-PCR detection. To increase the accuracy of the qRT-PCR, primers were designed with the forward primer crossing the back-splicing junction site. The expression patterns of the selected circRNAs according to the qPCR verification were consistent with the RNA sequencing results, except in the case of circSETD3-1 (Figure 4). CircGAS2-2, circFGFR2-2, circPEPD-1, and circGLI3-1 were significantly downregulated, whereas circSTK39 and circMAPKBP1-1 were significantly upregulated. These results indicate that the sequencing analysis was reliable.

### 3.5. Experimental Validation and Spatiotemporal Expression of circGAS2-2

**Figure 5:**
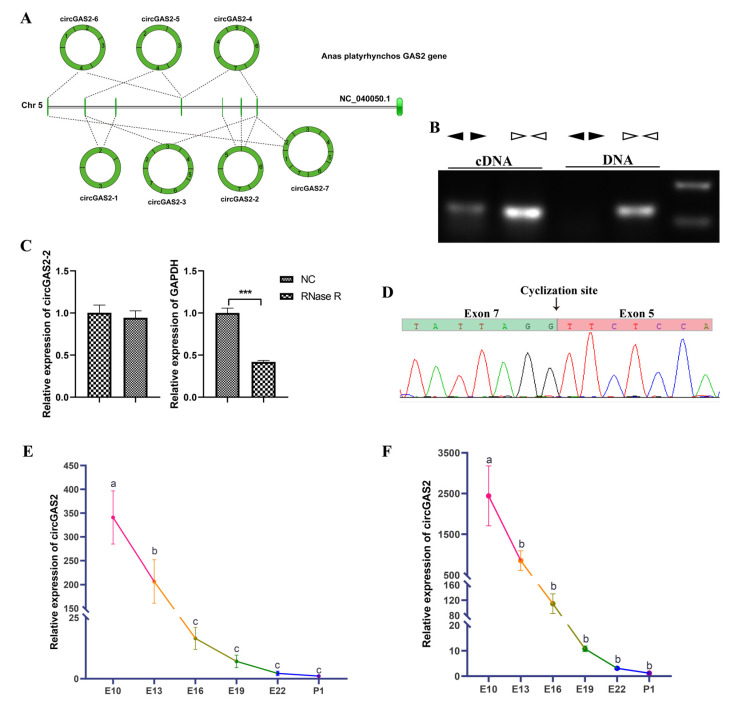
Validation and differential expression of circGAS2-2. (**A**) Scheme of the genomic structure and circular transcripts of *GAS2*. The green rectangles and the green vertical lines represent the exons of *GAS2*. The circular rings represent seven circRNAs derived from *GAS2*. (**B**) A divergent primer set amplified the fragment of circGAS2-2 in cDNA but not in genomic DNA. The black triangles represent the divergent primers, and the white triangles represent the convergent primers. (**C**) qRT-PCR results showing that RNase R had no impact on circGAS2-2. (**D**) Sanger sequencing results confirming the junction sequence of circGAS2-2. (**E**,**F**) Spatiotemporal expression of circGAS2-2 from E10 to P1 in breast muscle and leg muscle, respectively. The values are shown as means ± SEM. *** *p* < 0.001. The different letters indicate significant differences between the two groups (*p* < 0.05).

Owing to the abundant expression of downregulated circular RNAs, circGAS2-2 was selected as the candidate circRNA for the subsequent functional validation experiments in duck primary breast myoblasts. The genomic structure of the duck *GAS2* gene is shown in Figure 5A. The *GAS2* gene is located on duck chromosome 5 and contains eight exons. Interestingly, the seven circular RNAs derived from the *GAS2* gene based on our sequencing data were all exonic circular RNAs. Of these circular RNAs, circGAS2-2 had the highest expression level. A divergent and convergent primer set based on the duck *GAS2* genomic sequence was designed to confirm the sequence and junction site of circGAS2-2. The PCR procedure with the divergent and convergent primers was run using the cDNA and genomic DNA as templates. The electrophoresis results revealed that the convergent primer PCR products were amplified by both templates as expected, whereas no divergent primer PCR product was found with the genomic DNA template (Figure 5B). The RNase R treatment also confirmed the existence of circGAS2-2. The qRT-PCR data results demonstrated that RNase R had little effect on circGAS2-2; however, the RNA levels of *GAPDH*, which was used as a linear RNA control, were significantly decreased (Figure 5C). Furthermore, an analysis of the PCR products with the divergent primers by Sanger sequencing showed that the 5′ site of exon five and the 3′ site of exon seven were linked, indicating that circGAS2-2 is generated from the fifth, sixth, and seventh exons of *GAS2* (Figure 5D). The expression levels of circGAS2-2 on E10, E13, E16, E19, E22, and 1 day post-hatching (P1) were detected by qRT-PCR. The expression trend showed a gradual decline from E13 to P1 (Figure 5E,F). These findings demonstrate that circGAS2-2 exists and is expressed differently throughout embryonic skeletal muscle development, suggesting that circGAS2-2 may have key roles in duck muscle development.

### 3.6. circGAS2-2 Promotes the Proliferation of Duck Breast Primary Myoblasts

**Figure 6:**
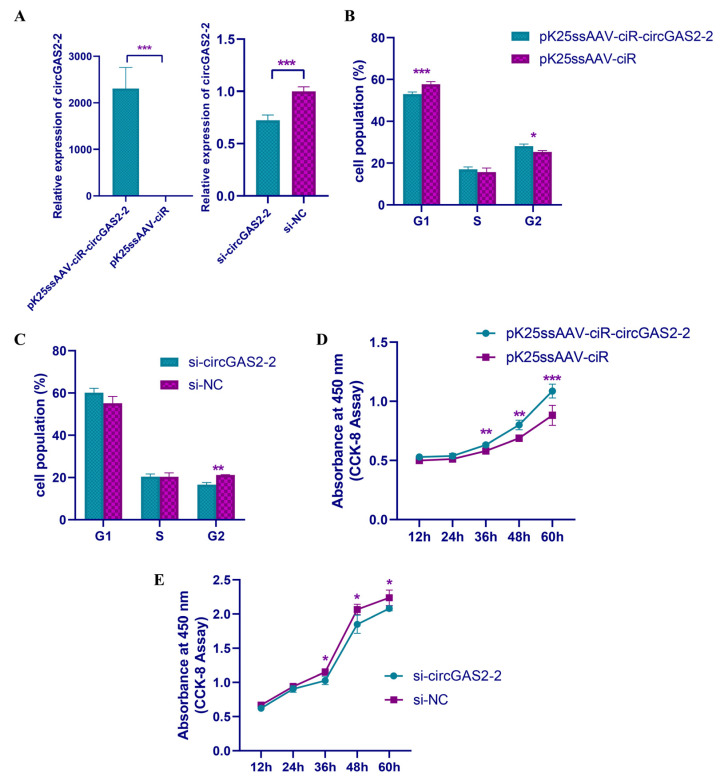
circGAS2-2 accelerates the cell cycle and promotes the proliferation of duck primary myoblasts. (**A**) Relative expression levels of circGAS2-2 after electroporation of myoblasts with pK25ssAAV-ciR-circGAS2-2/pK25ssAAV-ciR and si-circGAS2-2/siRNA NC for 48 h. (**B**) Analyses of the cell cycle after electroporation with pK25ssAAV-cirGAS2-2 or pK25ssAAV-cirGAS2-2 on duck primary myoblasts. (**C**) Analyses of the cell cycle after electroporation with si-circGAS2-2 or siRNA NC in duck primary myoblasts. (**D**,**E**) Cell-counting assay of duck primary myoblasts after electroporation with pK25ssAAV-ciR-circGAS2-2/pK25ssAAV-ciR and si-circGAS2-2/siRNA NC for 12 h, 24 h, 36 h, 48 h, and 60 h. The values are shown as means ± SEM. * *p* < 0.05; ** *p* < 0.01; *** *p* < 0.001.

Given that circGAS2-2 was differentially expressed at several phases of the duck breast muscle development, we hypothesized that it might regulate the proliferation and differentiation of breast primary myoblasts. To confirm our hypothesis, duck primary myoblasts were isolated, cultured, and induced to differentiate. To investigate the function of circGAS2-2, an overexpression vector and short interfering RNAs (siRNAs) were constructed and electroporated into myoblasts. The group with the overexpression vector expressed circGAS2-2 with high efficiency compared with the group electroporated with pK25ssAAV-ciR, and the siRNA significantly knocked down the level of circGAS2-2 compared with the siRNA negative control (NC) group (Figure 6A). The cell cycle in the primary myoblasts from the ducks was analyzed using flow cytometry after the electroporation. The results revealed that the overexpression of circGAS2-2 significantly enhanced cell cycling by decreasing the number of cells in the G1 phase and increasing the number in the G2 phase (Figure 6B). By contrast, knockdown of circGAS2-2 markedly reduced the cell population in the G2 stage and retarded the cell cycle of the primary myoblasts (Figure 6C). The duck primary myoblasts were tested for proliferation vitality using the cell-counting assay. The groups electroporated with the overexpression plasmid had markedly higher proliferation vitality than the pK25ssAAV-ciR group at 36 h, 48 h, and 60 h after the electroporation (Figure 6D). Conversely, the electroporation with si-circGAS2-2 significantly suppressed the cell growth rate compared with the siRNA NC (Figure 6E). Taken together, these results suggest that circGAS2-2 promotes the proliferation of duck breast primary myoblasts. We also investigated the function of circGAS2-2 in myoblast differentiation. After electroporation with pK25ssAAV-ciR-circGAS2-2/pK25ssAAV-ciR and si-circGAS2-2/siRNA NC, the expression levels of the myoblast differentiation marker genes, myogenic factor 5 (*MYF5*), *MRF4,* myogenin (*MYOG*), and myogenic differentiation 1 (*MYOD1*), were determined. The qRT-PCR results showed that neither ectopic expression nor knockdown of circGAS2-2 significantly changed the expression levels of *MYOD1*, *MRF4*, *MYF5,* or *MYOG* (Figure S3), indicating that circGAS2-2 had no regulatory effect on myoblast differentiation.

## 4. Discussion

Circular RNAs have been found to be abundant and dynamically expressed in different tissues of various species. Non-canonical RNA splicing was detected in large transcriptome sequencing data, indicating that circular RNAs are universal in the transcriptome [43]. When circular RNAs in mice were identified, their biological function became a focus of speculation [44]. Circular RNA sequencing databases for various domestic animals, including pigs, cattle, and sheep, have now been constructed [27,45,46]. However, few studies have considered circular RNAs in poultry [28,47,48]. In this study, we investigated the expression of circular RNAs in two stages of duck breast muscle development. A total of 16,622 circular RNAs were identified in the E13 and E19 groups, which was greater than the number detected in duck follicles (4204) and chicken embryonic leg muscles (13,377) [28,48]. These results demonstrate that circular RNAs are universally abundant and dynamically expressed in different tissues. The circular RNAs identified in this study were mainly derived from exons, consistent with studies in chicken and pig muscles but not with those in duck follicles, indicating that back-splicing of pre-mRNA is tissue-specific [27,28,48].

We identified 260 circular RNAs that were differentially expressed between the E13 and E19 groups. These differentially expressed circular RNAs were significantly enriched in GO terms associated with skeletal muscle cell differentiation and muscle tissue regeneration. A KEGG analysis showed that the circular RNAs were significantly enriched in the following: gap junctions, the calcium signaling pathway, purine metabolism, vascular smooth muscle contraction, the GnRH signaling pathway, the MAPK signaling pathway, and cardiac muscle contraction. Gap junction proteins are associated with cell communication in cardiac muscle cells [49]. Calcium homeostasis has important roles in controlling the fate of muscle tissue through multiple pathways, and calcium signaling has a vital regulatory function in duck breast muscle development [50,51]. Moreover, the MAPK and GnRH signaling pathways are associated with animal body weight and contribute to muscle growth [52,53]. According to the PPI analysis, many proteins encoded by the parental genes of the differentially expressed circular RNAs had interactions with other parental proteins. *FGFR2* is an important activator of satellite cells in skeletal muscle growth, and circFGFR2 promotes the proliferation and differentiation of primary myoblasts [2,54]. Taken together, these results indicate that differentially expressed circular RNAs may participate in skeletal muscle growth and development via regulation of their parental genes.

Circular RNAs, which play a major role in myogenesis by acting as sponges for miRNAs, have not yet been fully understood [29,55]. As a scaffold for miR-378a-3p, circLMO7 increases histone deacetylase 4 (*HDAC4*) expression, decreases myocyte enhancer factor 2A (*MEF2A*) expression, and promotes the differentiation of myoblasts [56]. Circular RNAs also regulate myogenesis by producing functional peptides [14]. Genes containing multiple exons usually generate different circular transcripts by alternative back-splicing [37]. In this study, many duck genes were found to transcribe several circular RNAs, most of which were circRNAs. A total of seven circRNAs were produced by the *GAS2* gene. We selected a circRNA derived from the duck *GAS2* gene for validation experiments in myoblasts, owing to its high expression levels among the downregulated circular RNAs. Before the myoblast experiments, we used divergent–convergent primer PCR, Sanger sequencing, and RNase R treatments to validate the presence of circGAS2-2. The results demonstrated that circGAS2-2 is abundantly expressed in duck breast muscle.

The growth arrest-specific (GAS) gene family has been linked to reversible growth arrest. The actin cytoskeleton and cell shape are swiftly reset by GAS2-regulated microfilament changes in response to growth arrest triggered by environmental stimuli, such as apoptosis, distinct proliferative stimulation, and varied growth factors [57]. The GAS2 protein is an apoptotic regulator that interacts with caspase-3 and caspase-7 and promotes the proliferation of T-cell acute lymphoblastic leukemia by activating the Wnt/β-catenin pathway [58,59,60]. The function of *GAS2* in muscle development has not yet been reported. In this study, we found that circGAS2-2 could promote the proliferation of duck breast primary myoblasts by facilitating the progression of the cell cycle. However, neither overexpression nor knockdown of circGAS2-2 had any significant effect on the expression levels of the myoblast differentiation-determining factors (*MYOD1*, *MYOG*, *MYF5*, and *MRF4*), indicating that circGAS2-2 does not regulate duck myoblast differentiation. Thus, further investigation is needed to determine the underlying mechanisms by which circGAS2-2 promotes myoblast proliferation.

## 5. Conclusions

In conclusion, our results showed that circular RNAs were abundantly and differentially expressed during the process of duck skeletal muscle development. The circular RNA circGAS2-2, which is derived from *GAS2*, could promote myoblast proliferation in ducks.

## Data Availability

All raw sequencing data and processing files are available in the GEO with accession number GSE152947 (https://www.ncbi.nlm.nih.gov/geo/query/acc.cgi?acc=GSE152947, to be released on 22 June 2023).

## Supplementary Materials

The following supporting information can be downloaded at: https://www.mdpi.com/article/10.3390/vetsci10020075/s1, Figure S1: PCR products of *CHD1* gene amplification; Figure S2: Venn diagrams of circular RNAs expressed in each sample from the same group; Figure S3: Expression of the myoblast differentiation-related gene after the overexpression and knockdown of circGAS2-2; Table S1: Primers and siRNA; Table S2: Mapping statistics; Table S3: The statistical result of the circular RNA identification; Table S4: The results of the GO enrichment; Table S5: The results of the KEGG enrichment analysis.

## References

[1] Scanes C.G., Harvey S., Marsh J.A., King D.B. (1984). Hormones and Growth in Poultry. Poult. Sci..

[2] Chen X., Ouyang H., Wang Z., Chen B., Nie Q. (2018). A Novel Circular RNA Generated by FGFR2 Gene Promotes Myoblast Proliferation and Differentiation by Sponging MiR-133a-5p and MiR-29b-1-5p. Cells.

[3] Braun T., Gautel M. (2011). Transcriptional Mechanisms Regulating Skeletal Muscle Differentiation, Growth and Homeostasis. Nat. Rev. Mol. Cell Biol..

[4] Abmayr S.M., Pavlath G.K. (2012). Myoblast Fusion: Lessons from Flies and Mice. Development.

[5] Buckingham M. (2001). Skeletal Muscle Formation in Vertebrates. Curr. Opin. Genet. Dev..

[6] Luo W., Wu H., Ye Y., Li Z., Hao S., Kong L., Zheng X., Lin S., Nie Q., Zhang X. (2014). The Transient Expression of MiR-203 and Its Inhibiting Effects on Skeletal Muscle Cell Proliferation and Differentiation. Cell Death Dis..

[7] Li Z., Cai B., Abdalla B.A., Zhu X., Zheng M., Han P., Nie Q., Zhang X. (2019). LncIRS1 Controls Muscle Atrophy via Sponging MiR-15 Family to Activate IGF1-PI3K/AKT Pathway. J. Cachexia Sarcopenia Muscle.

[8] Luo W., Chen J., Li L., Ren X., Cheng T., Lu S., Lawal R.A., Nie Q., Zhang X., Hanotte O. (2019). C-Myc Inhibits Myoblast Differentiation and Promotes Myoblast Proliferation and Muscle Fibre Hypertrophy by Regulating the Expression of Its Target Genes, MiRNAs and LincRNAs. Cell Death Differ..

[9] Memczak S., Jens M., Elefsinioti A., Torti F., Krueger J., Rybak A., Maier L., Mackowiak S.D., Gregersen L.H., Munschauer M. (2013). Circular RNAs Are a Large Class of Animal RNAs with Regulatory Potency. Nature.

[10] Rybak-Wolf A., Stottmeister C., Glažar P., Jens M., Pino N., Giusti S., Hanan M., Behm M., Bartok O., Ashwal-Fluss R. (2015). Circular RNAs in the Mammalian Brain Are Highly Abundant, Conserved, and Dynamically Expressed. Mol. Cell.

[11] Yuan X., Yuan Y., He Z., Li D., Zeng B., Ni Q., Yang M., Yang D. (2020). The Regulatory Functions of Circular RNAs in Digestive System Cancers. Cancers.

[12] Zhang P., Chao Z., Zhang R., Ding R., Wang Y., Wu W., Han Q., Li C., Xu H., Wang L. (2019). Circular RNA Regulation of Myogenesis. Cells.

[13] Jia E., Zhou Y., Liu Z., Wang L., Ouyang T., Pan M., Bai Y., Ge Q. (2020). Transcriptomic Profiling of Circular RNA in Different Brain Regions of Parkinson’s Disease in a Mouse Model. Int. J. Mol. Sci..

[14] Legnini I., Di Timoteo G., Rossi F., Morlando M., Briganti F., Sthandier O., Fatica A., Santini T., Andronache A., Wade M. (2017). Circ-ZNF609 Is a Circular RNA That Can Be Translated and Functions in Myogenesis. Mol. Cell.

[15] Pamudurti N.R., Bartok O., Jens M., Ashwal-Fluss R., Stottmeister C., Ruhe L., Hanan M., Wyler E., Perez-Hernandez D., Ramberger E. (2017). Translation of CircRNAs. Mol. Cell.

[16] Chen L.-L. (2020). The Expanding Regulatory Mechanisms and Cellular Functions of Circular RNAs. Nat. Rev. Mol. Cell Biol..

[17] Li Z., Huang C., Bao C., Chen L., Lin M., Wang X., Zhong G., Yu B., Hu W., Dai L. (2015). Exon-Intron Circular RNAs Regulate Transcription in the Nucleus. Nat. Struct. Mol. Biol..

[18] Liu Y., Su H., Zhang J., Liu Y., Feng C., Han F. (2020). Back-Spliced RNA from Retrotransposon Binds to Centromere and Regulates Centromeric Chromatin Loops in Maize. PLoS Biol..

[19] Salmena L., Poliseno L., Tay Y., Kats L., Pandolfi P.P. (2011). A CeRNA Hypothesis: The Rosetta Stone of a Hidden RNA Language?. Cell.

[20] Du W.W., Yang W., Liu E., Yang Z., Dhaliwal P., Yang B.B. (2016). Foxo3 Circular RNA Retards Cell Cycle Progression via Forming Ternary Complexes with P21 and CDK2. Nucleic Acids Res..

[21] Zeng Y., Du W.W., Wu Y., Yang Z., Awan F.M., Li X., Yang W., Zhang C., Yang Q., Yee A. (2017). A Circular RNA Binds To and Activates AKT Phosphorylation and Nuclear Localization Reducing Apoptosis and Enhancing Cardiac Repair. Theranostics.

[22] Qin T., Li J., Zhang K.-Q. (2020). Structure, Regulation, and Function of Linear and Circular Long Non-Coding RNAs. Front. Genet..

[23] Jeck W.R., Sorrentino J.A., Wang K., Slevin M.K., Burd C.E., Liu J., Marzluff W.F., Sharpless N.E. (2013). Circular RNAs Are Abundant, Conserved, and Associated with ALU Repeats. RNA.

[24] Dori M., Bicciato S. (2019). Integration of Bioinformatic Predictions and Experimental Data to Identify CircRNA-MiRNA Associations. Genes.

[25] Shen X., Liu Z., Cao X., He H., Han S., Chen Y., Cui C., Zhao J., Li D., Wang Y. (2019). Circular RNA Profiling Identified an Abundant Circular RNA CircTMTC1 That Inhibits Chicken Skeletal Muscle Satellite Cell Differentiation by Sponging MiR-128-3p. Int. J. Biol. Sci..

[26] Zhang P., Xu H., Li R., Wu W., Chao Z., Li C., Xia W., Wang L., Yang J., Xu Y. (2018). Assessment of Myoblast Circular RNA Dynamics and Its Correlation with MiRNA during Myogenic Differentiation. Int. J. Biochem. Cell Biol..

[27] Liang G., Yang Y., Niu G., Tang Z., Li K. (2017). Genome-Wide Profiling of Sus Scrofa Circular RNAs across Nine Organs and Three Developmental Stages. DNA Res..

[28] Ouyang H., Chen X., Wang Z., Yu J., Jia X., Li Z., Luo W., Abdalla B.A., Jebessa E., Nie Q. (2017). Circular RNAs Are Abundant and Dynamically Expressed during Embryonic Muscle Development in Chickens. DNA Res..

[29] Wang Y., Li M., Wang Y., Liu J., Zhang M., Fang X., Chen H., Zhang C. (2019). A Zfp609 Circular RNA Regulates Myoblast Differentiation by Sponging MiR-194-5p. Int. J. Biol. Macromol..

[30] Gu L.H., Xu T.S., Huang W., Xie M., Shi W.B., Sun S.D., Hou S.S. (2013). Developmental Characteristics of Pectoralis Muscle in Pekin Duck Embryos. Genet. Mol. Res..

[31] Chen B., Liu S., Zhang W., Xiong T., Zhou M., Hu X., Mao H., Liu S. (2022). Profiling Analysis of N6-Methyladenosine MRNA Methylation Reveals Differential M6A Patterns during the Embryonic Skeletal Muscle Development of Ducks. Animals.

[32] Li H., Hu Y., Song C., Ji G., Liu H., Xu W., Ding J. (2015). A New Primer for Sex Identification of Ducks and a Minimally Invasive Technique for Sampling of Allantoic Fluid to Detect Sex during Bird Embryo Development. Sex. Dev..

[33] Kechin A., Boyarskikh U., Kel A., Filipenko M. (2017). CutPrimers: A New Tool for Accurate Cutting of Primers from Reads of Targeted Next Generation Sequencing. J. Comput. Biol..

[34] Langmead B., Salzberg S.L. (2012). Fast Gapped-Read Alignment with Bowtie 2. Nat. Methods.

[35] Kim D., Pertea G., Trapnell C., Pimentel H., Kelley R., Salzberg S.L. (2013). TopHat2: Accurate Alignment of Transcriptomes in the Presence of Insertions, Deletions and Gene Fusions. Genome Biol..

[36] Kim D., Salzberg S.L. (2011). TopHat-Fusion: An Algorithm for Discovery of Novel Fusion Transcripts. Genome Biol..

[37] Zhang X.-O., Dong R., Zhang Y., Zhang J.-L., Luo Z., Zhang J., Chen L.-L., Yang L. (2016). Diverse Alternative Back-Splicing and Alternative Splicing Landscape of Circular RNAs. Genome Res..

[38] Gao Y., Wang J., Zhao F. (2015). CIRI: An Efficient and Unbiased Algorithm for de Novo Circular RNA Identification. Genome Biol..

[39] Zhang X.-O., Wang H.-B., Zhang Y., Lu X., Chen L.-L., Yang L. (2014). Complementary Sequence-Mediated Exon Circularization. Cell.

[40] Robinson M.D., McCarthy D.J., Smyth G.K. (2010). EdgeR: A Bioconductor Package for Differential Expression Analysis of Digital Gene Expression Data. Bioinformatics.

[41] Huang D.W., Sherman B.T., Lempicki R.A. (2009). Systematic and Integrative Analysis of Large Gene Lists Using DAVID Bioinformatics Resources. Nat. Protoc..

[42] Szklarczyk D., Gable A.L., Nastou K.C., Lyon D., Kirsch R., Pyysalo S., Doncheva N.T., Legeay M., Fang T., Bork P. (2020). The STRING Database in 2021: Customizable Protein–Protein Networks, and Functional Characterization of User-Uploaded Gene/Measurement Sets. Nucleic Acids Res..

[43] Salzman J., Gawad C., Wang P.L., Lacayo N., Brown P.O. (2012). Circular RNAs Are the Predominant Transcript Isoform from Hundreds of Human Genes in Diverse Cell Types. PLoS One.

[44] Guo J.U., Agarwal V., Guo H., Bartel D.P. (2014). Expanded Identification and Characterization of Mammalian Circular RNAs. Genome Biol..

[45] Li C., Li X., Ma Q., Zhang X., Cao Y., Yao Y., You S., Wang D., Quan R., Hou X. (2017). Genome-Wide Analysis of Circular RNAs in Prenatal and Postnatal Pituitary Glands of Sheep. Sci. Rep..

[46] Zhang C., Wu H., Wang Y., Zhu S., Liu J., Fang X., Chen H. (2016). Circular RNA of Cattle Casein Genes Are Highly Expressed in Bovine Mammary Gland. J. Dairy Sci..

[47] Shen M., Li T., Zhang G., Wu P., Chen F., Lou Q., Chen L., Yin X., Zhang T., Wang J. (2019). Dynamic Expression and Functional Analysis of CircRNA in Granulosa Cells during Follicular Development in Chicken. BMC Genom..

[48] Wu Y., Xiao H., Pi J., Zhang H., Pan A., Pu Y., Liang Z., Shen J., Du J. (2020). The Circular RNA Aplacirc_13267 Upregulates Duck Granulosa Cell Apoptosis by the Apla-MiR-1-13/THBS1 Signaling Pathway. J. Cell. Physiol..

[49] Ghasemi Tahrir F., Gupta M., Myers V., Gordon J., Cheung J.Y., Feldman A.M., Khalili K. (2019). Role of Bcl2-Associated Athanogene 3 in Turnover of Gap Junction Protein, Connexin 43, in Neonatal Cardiomyocytes. Sci. Rep..

[50] Damm T.B., Egli M. (2014). Calcium’s Role in Mechanotransduction during Muscle Development. Cell. Physiol. Biochem..

[51] Xu T., Gu L., Schachtschneider K.M., Liu X., Huang W., Xie M., Hou S. (2014). Identification of Differentially Expressed Genes in Breast Muscle and Skin Fat of Postnatal Pekin Duck. PLoS One.

[52] Widmann P., Reverter A., Fortes M.R.S., Weikard R., Suhre K., Hammon H., Albrecht E., Kuehn C. (2013). A Systems Biology Approach Using Metabolomic Data Reveals Genes and Pathways Interacting to Modulate Divergent Growth in Cattle. BMC Genom..

[53] Xie S.-J., Li J.-H., Chen H.-F., Tan Y.-Y., Liu S.-R., Zhang Y., Xu H., Yang J.-H., Liu S., Zheng L.-L. (2018). Inhibition of the JNK/MAPK Signaling Pathway by Myogenesis-Associated MiRNAs Is Required for Skeletal Muscle Development. Cell Death Differ..

[54] Ghanim H., Dhindsa S., Batra M., Green K., Abuaysheh S., Kuhadiya N.D., Makdissi A., Chaudhuri A., Dandona P. (2019). Effect of Testosterone on FGF2, MRF4, and Myostatin in Hypogonadotropic Hypogonadism: Relevance to Muscle Growth. J. Clin. Endocrinol. Metab..

[55] Ouyang H., Chen X., Li W., Li Z., Nie Q., Zhang X. (2018). Circular RNA CircSVIL Promotes Myoblast Proliferation and Differentiation by Sponging MiR-203 in Chicken. Front. Genet..

[56] Wei X., Li H., Yang J., Hao D., Dong D., Huang Y., Lan X., Plath M., Lei C., Lin F. (2017). Circular RNA Profiling Reveals an Abundant CircLMO7 That Regulates Myoblasts Differentiation and Survival by Sponging MiR-378a-3p. Cell Death Dis..

[57] Zhang N., Zhao C., Zhang X., Cui X., Zhao Y., Yang J., Gao X. (2021). Growth Arrest–Specific 2 Protein Family: Structure and Function. Cell Prolif..

[58] Kong Y., Zhao S., Tian H., Hai Y. (2020). GAS2 Promotes Cell Proliferation and Invasion and Suppresses Apoptosis in Pediatric T-Cell Acute Lymphoblastic Leukemia and Activates Wnt/β-Catenin Pathway. OncoTargets Ther..

[59] Zhu R.-X., Cheng A.S.L., Chan H.L.Y., Yang D.-Y., Seto W.-K. (2019). Growth Arrest-Specific Gene 2 Suppresses Hepatocarcinogenesis by Intervention of Cell Cycle and P53-Dependent Apoptosis. World J. Gastroenterol..

[60] Sgorbissa A., Benetti R., Marzinotto S., Schneider C., Brancolini C. (1999). Caspase-3 and Caspase-7 but Not Caspase-6 Cleave Gas2 in Vitro: Implications for Microfilament Reorganization during Apoptosis. J. Cell Sci..

