# Supplementary material for: Transcriptome RNA Sequencing Reveals That Circular RNAs Are Abundantly Expressed in Embryonic Breast Muscle of Duck
Source: Vet Sci. 2023 Jan 19;10(2):75. doi: 10.3390/vetsci10020075 (PMC10004440; doi:10.3390/vetsci10020075)
Supplement: Supplementary file 1 [file vetsci-10-00075-s001.zip › Supplementary Figures.pptx]

## Slide 1
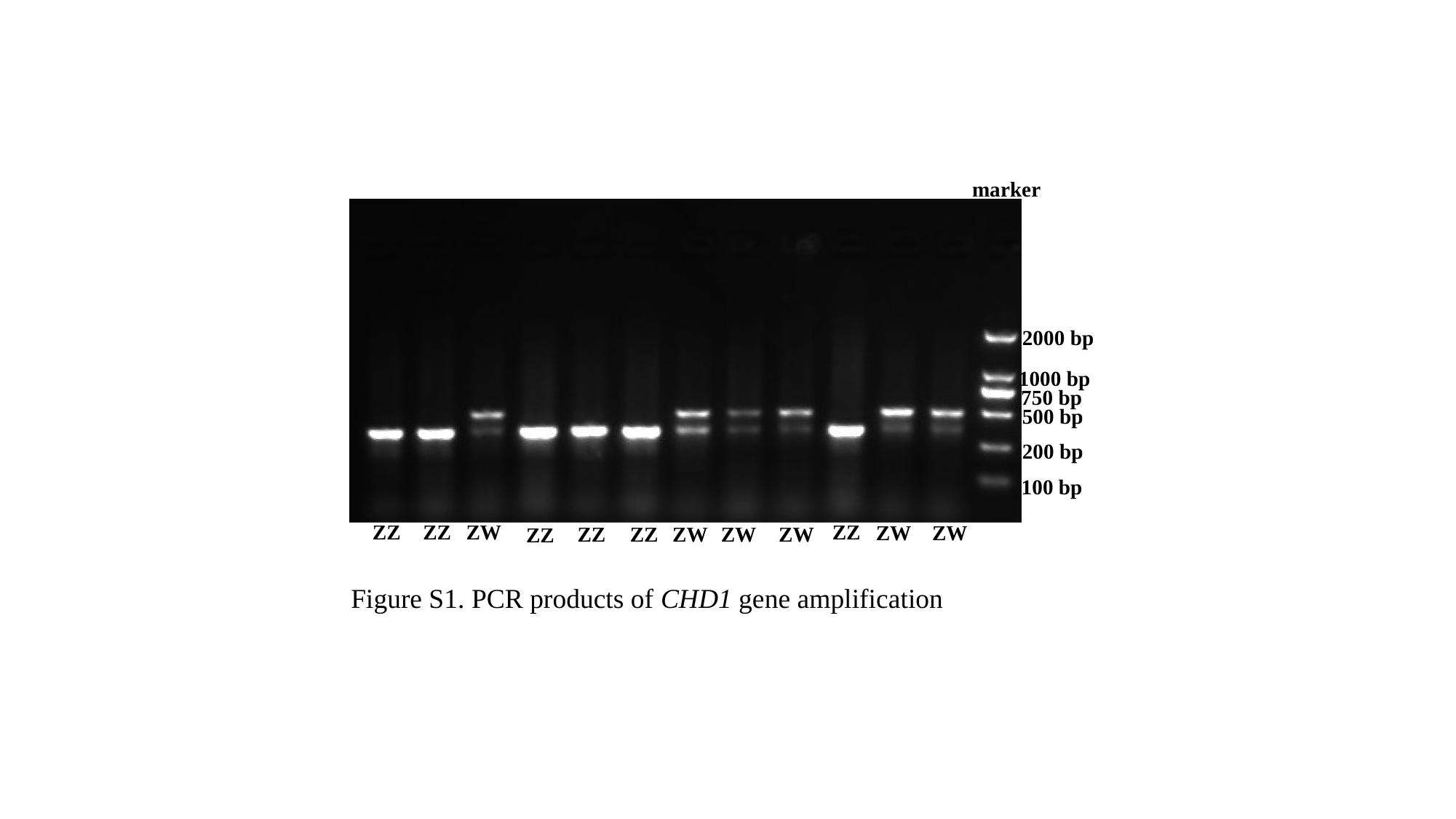

marker
2000 bp
1000 bp
750 bp
500 bp
200 bp
100 bp
ZW
ZZ
ZZ
ZZ
ZW
ZW
ZW
ZW
ZW
ZZ
ZZ
ZZ
Figure S1. PCR products of CHD1 gene amplification

## Slide 2
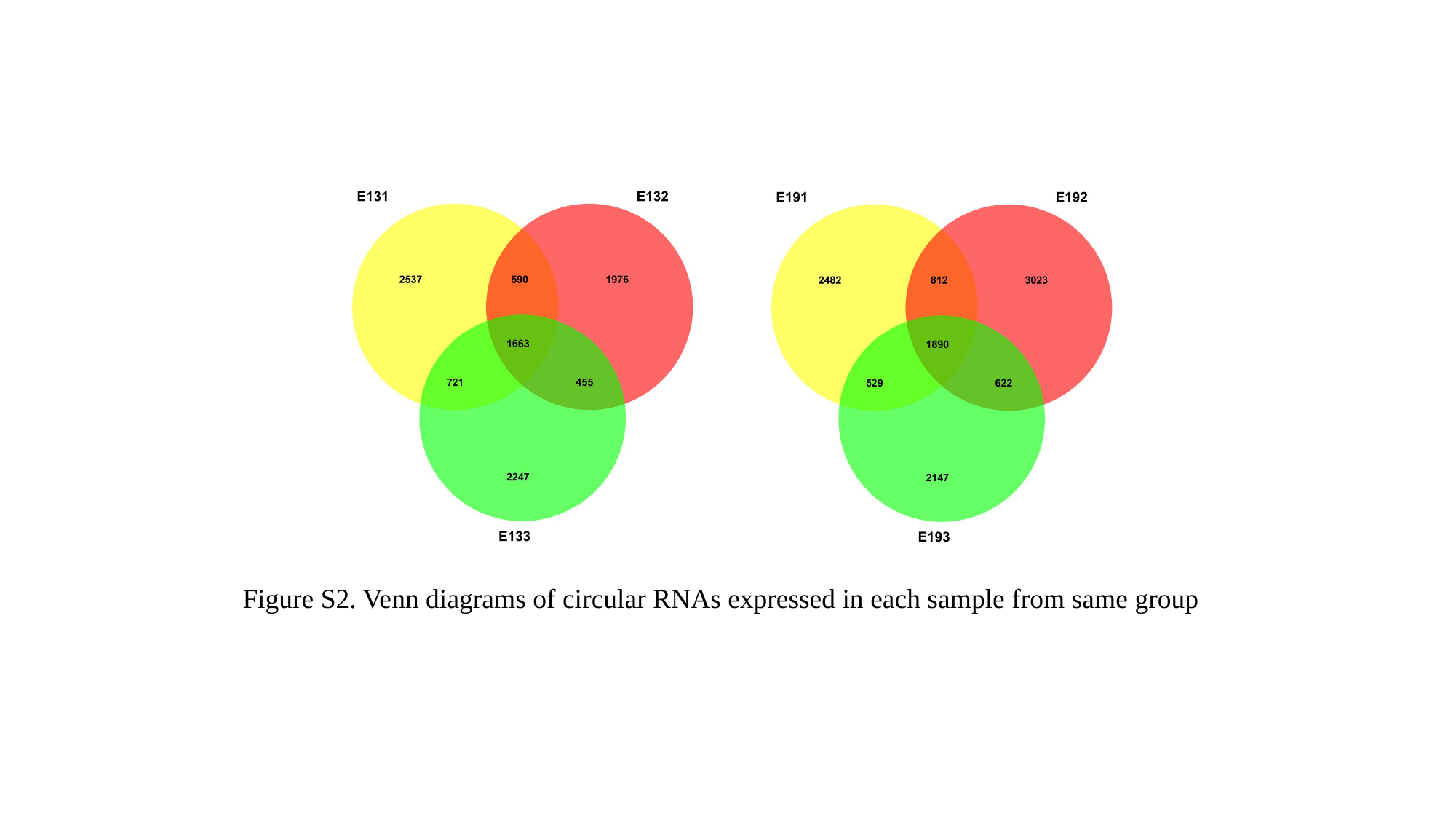

Figure S2. Venn diagrams of circular RNAs expressed in each sample from same group

## Slide 3
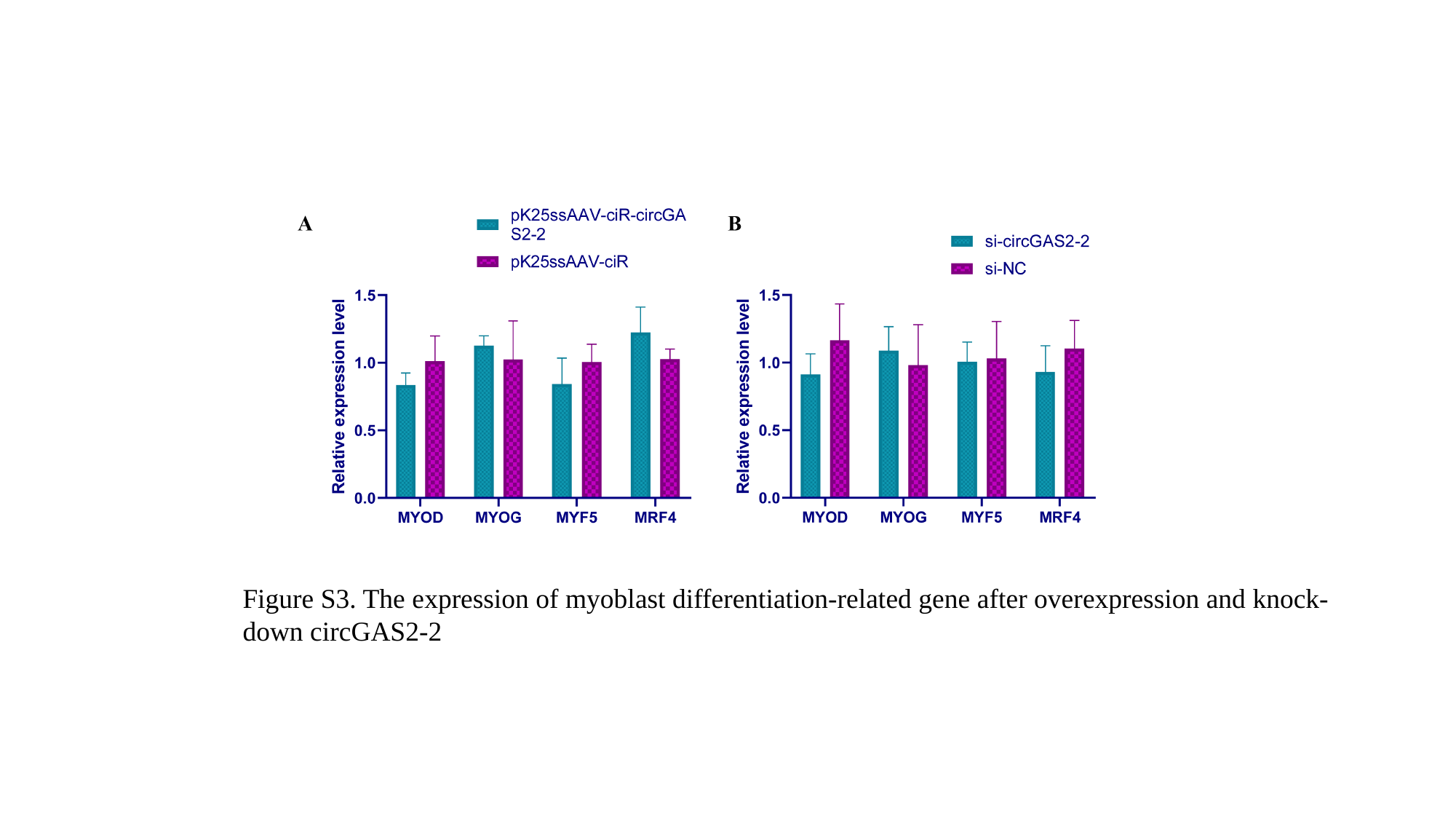

Figure S3. The expression of myoblast differentiation-related gene after overexpression and knock-down circGAS2-2
