# Supplementary material for: Transcriptome RNA Sequencing Reveals That Circular RNAs Are Abundantly Expressed in Embryonic Breast Muscle of Duck
Source: Vet Sci. 2023 Jan 19;10(2):75. doi: 10.3390/vetsci10020075 (PMC10004440; doi:10.3390/vetsci10020075)
Supplement: Supplementary file 1 [file vetsci-10-00075-s001.zip › Table S1.pdf]

Supplementary Table S1. Primers and siRNA

Name	Sequence (5’ - 3’)	Anneal temperature (°C)	Product size (bp)
HPF	AGTGCATTGCAGAAGCAATATT	55	495 or 351
HPR	GCCTCCTGTTTATTATAGAATTCAT		
qcircGAS2-2-F4	AGATCCTCTTTATTAGGTTCTCC	62	237
qcircGAS2-2-R4	ATGTGTTTAACAGCATCATCC		
circFGFR2-F1	GACAGCAACCAGATGAAGCCG	58	82
circFGFR2-R1	CAAGTGGTTCTCCTGGAAGTG		
qcircSTK39-F1	CTTGTCTTAAGATTAAGGTTAG	60	116
qcircSTK39-F1	GCCTTCCTCACTCTTCTCATC		
qcircPEPD-F1	AATATGAGCTGGAGAGGATTC	58	83
qcircPEPD-R1	TATCTCAGTCACGTAGTGGAC		
qcircMAPKBP1-F1	GTCCCCGGCTCTCAGTGAGT	58	134
qcircMAPKBP1-R1	GACGACTGCTGCTGTAATCT		
qcircGLI3-F1	GGAACAGCTAGTGCATCCCTG	58	148
qcircGLI3-R1	GTTGGAGCAGGATGGATGAGA		
circSETD3-con-F1	CATTGGCATTCCACTTACT	58	191
circSETD3-con-R1	ATAGGCATACTGTCGAGCT		
circGAS2-2-con-F1	GGTGGAGCCTCCTGGATT	55.3	108
circGAS2-2-con-R1	TTCCCTGGAGACTTTGTTGG		
circGAS2-2-div-F	CACCTTGTAAATGCCCTAATAAA	60	102
circGAS2-2-div-R	GGCTGCTTGTGGAGAACCT		
qMYOD-F1	CGGCTCAGCAAGGTCAAC	60	114
qMYOD-R1	CTCGATGTAGCGGATGGC		
qMYOG-F1	TCACCTCCTGCCTGACGA	60	80
qMYOG-R1	CCTCTACGGCGATGCTCT		
qMYF5-F1	TGAACCAGGCGTTTGAGA	60	152
qMYF5-R1	CGGCAGGTGATAGTAGTTTTCT		
qMYF6-F1	CTGGAGATGGCGGAGGGT	60	81
qMYF6-R1	CTCTGGCGGCATTTGGTC		
si-circGAS2-2	Target sequence: TTATTAGGTTCTCCACAAG	/	/
