# Supplementary material for: Transcriptome RNA Sequencing Reveals That Circular RNAs Are Abundantly Expressed in Embryonic Breast Muscle of Duck
Source: Vet Sci. 2023 Jan 19;10(2):75. doi: 10.3390/vetsci10020075 (PMC10004440; doi:10.3390/vetsci10020075)
Supplement: Supplementary file 1 [file vetsci-10-00075-s001.zip › Table S2.pdf]

Supplementary Table S2. Mapping statistic

	E131	E132	E133	E191	E192	E193
Valid reads	51,399,992	43,390,748	48,858,000	48,304,568	49,988,422	48,189,728
Mapped reads	27,857,433 (54.20%)	24,558,167 (56.60%)	26,404,501 (54.04%)	26,761,498 (55.40%)	26,108,554 (52.23%)	27,662,245 (57.40%)
Unique Mapped reads	27,552,795 (53.60%)	24,268,221 (55.93%)	26,092,997 (53.41%)	26,447,449 (54.75%)	25,775,165 (51.56%)	27,393,795 (56.85%)
Multi Mapped reads	304,638 (0.59%)	289,946 (0.67%)	311,504 (0.64%)	314,049 (0.65%)	333,389 (0.67%)	268,450 (0.56%)
PE Mapped reads	12,514,034 (24.35%)	11,022,423 (25.40%)	11,859,845 (24.27%)	12,072,227 (24.99%)	11,731,441 (23.47%)	12,459,905 (25.86%)
Reads map to sense strand	13,780,195 (26.81%)	12,135,538 (27.97%)	13,048,112 (26.71%)	13,224,075 (27.38%)	12,889,385 (25.78%)	13,696,599 (28.42%)
Reads map to antisense strand	13,772,600 (26.79%)	12,132,683 (27.96%)	13,044,885 (26.70%)	13,223,374 (27.37%)	12,885,780 (25.78%)	13,697,196 (28.42%)
Non-splice reads	22,385,242 (43.55%)	20,259,887 (46.69%)	21,585,396 (44.18%)	21,713,828 (44.95%)	20,791,827 (41.59%)	22,893,856 (47.51%)
Splice reads	5,167,553 (10.05%)	4,008,334 (9.24%)	4,507,601 (9.23%)	4,733,621 (9.80%)	4,983,338 (9.97%)	4,499,939 (9.34%)
Unmapped reads	23,542,559 (45.80%)	18,832,581 (43.40%)	22,453,499 (45.96%)	21,543,070 (44.60%)	23,879,868 (47.77%)	20,527,483 (42.60%)

PE Mapped Reads: Pair-end mapped reads.
