# Supplementary material for: Transcriptome RNA Sequencing Reveals That Circular RNAs Are Abundantly Expressed in Embryonic Breast Muscle of Duck
Source: Vet Sci. 2023 Jan 19;10(2):75. doi: 10.3390/vetsci10020075 (PMC10004440; doi:10.3390/vetsci10020075)
Supplement: Supplementary file 1 [file vetsci-10-00075-s001.zip › Table S3.pdf]

Supplementary Table S3. The statistical result of circular RNAs identification

	**E131**	**E132**	**E133**	**E191**	**E192**	**E193**
Candidate back-spliced junction reads	846,164 (1.65%)	580,378 (1.34%)	710,651 (1.45%)	669,104 (1.39%)	860,613 (1.72%)	617,843 (1.28%)
Confident post reads	37,069 (0.07%)	22,829 (0.05%)	28,851 (0.06%)	35,361 (0.07%)	43,591 (0.09%)	30,107 (0.06%)
CircRNA number	3,354	2,903	3,118	3,433	3,833	3,245
CircRNA-hosting gene number	1,866	1,676	1,765	1,822	1,981	1,772
